# The m.3243A>G variant affects not only islet, hair, or retinal ganglion cells, but all cells

**DOI:** 10.3205/oc000251

**Published:** 2025-05-02

**Authors:** Josef Finsterer

**Affiliations:** 1 Neurology & Neurophysiology Center, Vienna, Austria

**Keywords:** mtDNA, m.3243A>G, MELAS, retinopathy, maternally inherited diabetes and deafness

## Letter to the editor

I read with interest Chwiejczak et al.’s article on a 47-year-old female with maternally-inherited diabetes and deafness (MIDD) due to the variant m.3243A>G which was also phenotypically manifested by progressive retinopathy [1]. Ophthalmologic examinations revealed visual impairment, pigmentary retinopathy in the macular and peripapillary areas, retinal atrophy, mottled appearance, hypoautofluorescence, attenuation of outer retinal layers, thinning of the deep capillary plexus, and decreased ERG responses [1]. The study is excellent, but some points need discussion.

The first point is that heteroplasmy rates have not been determined in affected or unaffected tissues [1]. Since heteroplasmy rates strongly influence the clinical presentation, it is essential to know them, not only to assess the disease progression and prognosis, but also for genetic counselling. 

A second point is that the mother or other first-degree relatives have not undergone ophthalmological or genetic examination. In order to assess whether the causative variant m.3243A>G was inherited from the mother or occurred sporadically, it is important to know whether the mother, her siblings, or her children were also carriers of the causative variant, despite negative family history. In approximately 75% of cases, mtDNA variants are maternally transmitted [2], making it very likely that the index patient’s variant was also inherited. The absence of phenotypic expression does not exclude that the mother was a carrier of the variant. 

The third point is that the index patient was not systematically evaluated for multisystem disease. To assess whether the index patient had maternally inherited diabetes and deafness (MIDD) only or MIDD plus, it is important to know whether organs other than the pancreas, ears and eyes were clinically or subclinically affected [3]. Since the index patient had non-diabetic retinopathy, she must be classified as MIDD plus. We should know whether the index patient was systematically screened for multiorgan disease. Other features of MIDD plus include myoclonus, myopathy, polyneuropathy, or impairment of the vestibule-ocular reflex [4]. 

The fourth point is that no results of cerebral magnetic resonance imaging (MRI) were provided. In particular, did she manifest in the brain with stroke-like episodes (SLEs), seizures, cognitive decline, ataxia, white matter lesions (WMLs), atrophy, calcifications, cerebral atrophy, and elevated cerebrospinal fluid (CSF) lactate, as has been previously reported [4].

The fifth point is that the quality of diabetes control has not been reported [1]. Therefore, we should know the serum levels of HbA1c, C-peptide, glutamic acid decarboxylase (GAD) antibodies, islet cell antibodies, and the antidiabetic treatment. Knowing HbA1c levels is crucial for assessing whether diabetes contributed to retinopathy and was responsible for vascular changes also in brain. 

A sixth point is that delayed P100 may be due not only to ocular dysfunction but also to cerebral dysfunction. For this reason it is essential to perform cerebral MRI to rule out that the delayed P100 has a central nervous system cause.

The diagnosis of familial lipodystrophy suggests that not only the index patient but also other first degree family members were carriers of the m.3243A>G variant. If a first-degree relative was diagnosed with lipodystrophy or diabetes, were they tested for the pathogenic variant? 

In summary, the excellent study has limitations that should be addressed before final conclusions are drawn. Carriers of the m.3243A>G variant should be prospectively examined for multisystem disease since the MT-TL variant can manifest clinically or subclinically in any organ. 

## Notes

### Author’s ORCID

Josef Finsterer: 0000-0003-2839-7305

### Competing interests

The author declares that he has no competing interests.
